# Supplementary material for: The Impact of Maternal Probiotics on Intestinal Vitamin D Receptor Expression in Early Life
Source: Biomolecules. 2023 May 16;13(5):847. doi: 10.3390/biom13050847 (PMC10216467; doi:10.3390/biom13050847)
Supplement: Supplementary file 1 [file biomolecules-13-00847-s001.zip › biomolecules-2267827-supplementary.pdf]

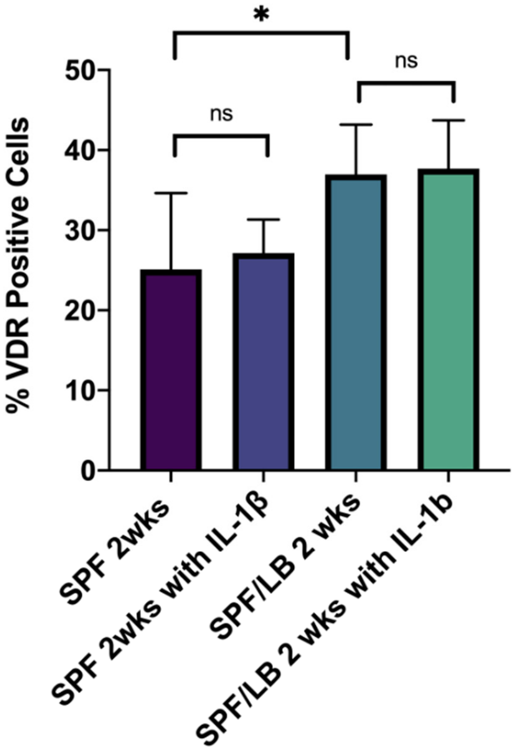

(a)

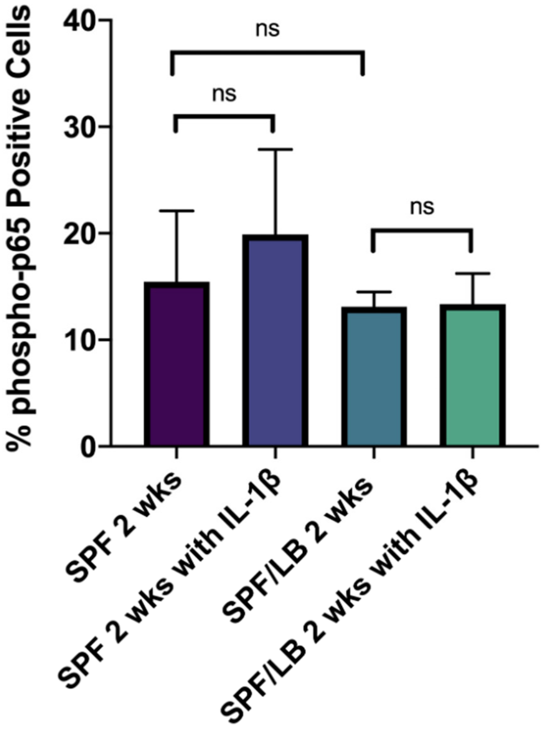

(b)

Supplemental Figure S1: (a) colonic VDR expression in: 2-week-old SPF and SPF with IL-1β mice. ns denotes non-significance with p-value = 0.59; 2-week-old SPF/LB and SPF/LB with IL-1β mice. ns denotes non-significance with p-value = 0.83; 2-week-old SPF and SPF/LB mice. * denotes statistical significance with p-value = 0.01; (b) colonic phospho-p65 expression in: 2-week-old SPF and SPF with IL-1β mice. ns denotes non-significance with p-value 0.24; 2-week-old SPF/LB and SPF/LB with IL-1β mice. ns denotes non-significance with p-value 0.87; 2-week-old SPF and SPF/LB mice. ns denotes non-significance with p-value = 0.40. n $\geq$ 3 for all subgroups
